# Movable Kidney, and Other Displacements and Malformations

**Published:** 1909-09

**Authors:** 


**Movable Kidney, and Other Displacements and Malformations.** By DAVID NEWMAN, M.D. Pp. vii, 233. London: Longmans, Green and Co. 1907.

The subject of movable kidney is here systematically dealt with, chiefly upon the basis of the author's personal experience. The normal and pathological anatomy of the kidney is first considered, especially with respect to the anatomical arrangements by which the kidney is retained in position.

In the clinical account of movable kidney the cases are classified, and illustrative examples of the various types of the affection are freely quoted. It is remarkable that a large proportion of the author's cases presented symptoms of definite disarrangement of the renal function, this proportion being in excess of the proportion usually met with in our experience.

For the successful fixation of movable kidney by operation it is considered essential to produce a "considerable granulating mass" between the surface of the kidney and the deeper parts of the wound; and it is therefore advised that the space in this situation be packed with gauze, which is left *in situ* for nine or ten days. Many surgeons find such a procedure quite unnecessary.

One specially interesting case is recorded, in which a true meso-nephron was found to exist at the operation for fixation, and perhaps this is the only case of the kind on record.

The well-illustrated chapter on malformations of the kidney is of great interest.

The book as a whole is an excellent record of most careful clinical observation and ripe opinion.

